# The interplay between thrombosis, stress, and social risk factors in trans persons on gender affirming hormone therapy

**DOI:** 10.1016/j.rpth.2026.103369

**Published:** 2026-01-23

**Authors:** Kevin Liang, Carl G. Streed, Pablo Buitron de la Vega, Beth M. Cohen

**Affiliations:** 1 Department of Medicine, Boston University Chobanian & Avedisian School of Medicine, Boston Medical Center, Boston, Massachusetts, USA; 2 Section of General Internal Medicine, Department of Medicine, Boston University Chobanian & Avedisian School of Medicine, Boston Medical Center, Boston, Massachusetts, USA; 3 GenderCare Center, Boston Medical Center, Boston, Massachusetts, USA; 4 Section of Endocrinology, Diabetes, Nutrition & Weight Management, Department of Medicine, Boston University Chobanian & Avedisian School of Medicine, Boston Medical Center, Boston, Massachusetts, USA

**Keywords:** health equity, hormones, social determinants of health, thrombophilia, thrombosis, transgender persons, venous thromboembolism

## Abstract

Although the use of exogenous hormone therapy has been the primary focus of transgender and gender diverse (trans) health research, additional factors that contribute to thrombosis warrant further evaluation in this population. Because stress is a trigger of hypercoagulability, disparities in thrombosis experienced by trans persons should be examined through a minority stress lens. Given the high burden of socioeconomic disparities experienced by trans persons and the potential moderating effect of socioeconomic status on minority stress, clinicians and researchers should assess the role of social risk factors in exacerbating stress and thrombosis in this population. Clotting during mental stress is thought to have evolved, in part, as an adaptive response in anticipation of bleeding from injury, acting via 2 hormonal pathways: catecholamine- and glucocorticoid-mediated pathways. Because trans persons face significant socioeconomic disparities, the theorized role of social risk factors in exacerbating stress-induced hypercoagulability should be studied in this population. This is especially relevant for those on gender affirming hormone therapy, which can have compounding prothrombotic effects. We define sex via the genetic complement of chromosomes, including cellular and molecular differences, as well as physical traits such as genitalia and reproductive organs. We define gender as comprising the social, environmental, cultural, and behavioral factors that influence a person's self-identity, health, and well-being.

## 1 Introduction

Transgender and gender diverse (trans) people experience worse health outcomes compared with their cisgender peers [1]. Aside from mental health conditions, venous thromboembolism (VTE) is perhaps the most studied diagnosis in trans populations. Given their known thrombotic effects in cisgender populations [2], the use of exogenous hormone therapy has been the primary focus of research in trans populations [3]. However, additional factors that contribute to thrombosis warrant further evaluation in trans people. Data from the TransPop study, the first national probability sample of the U.S. transgender population, revealed that transgender people had 3 times the odds of VTE than cisgender people (adjusted odds ratio [aOR], 3.35), with the difference between transgender and cisgender women (aOR, 3.94) driving this finding [4]. While a history of hormone use had a statistically nonsignificant association with higher VTE odds (aOR, 1.49 [95% CI: 0.21, 10.78]), transgender people with a history of hormone use had lower odds of any cardiovascular disease (CVD; aOR, 0.69 [95% CI: 0.24, 2.00]) compared with those without hormone use [4]. This suggests that hormone therapy—presumably by countering the psychological distress of gender dysphoria—may confer protection against allostatic load. Furthermore, other transgender-specific factors beyond hormone therapy may contribute to CVD risk. Although discrimination and psychological distress in this study were found not to be associated with VTE in trans people, they were significant predictors of current smoking [4], a well-established risk factor of hypercoagulability, which has a dramatic multiplicative effect on VTE risk when paired with combined oral contraceptives [5].

Many prospective studies have identified associations between psychosocial stress (ie, acute psychological stress, work stress, caregiving stress, depression, anxiety, and posttraumatic stress disorder) and an increased incidence of arterial thrombotic events, namely myocardial infarction, ischemic stroke, and acute limb ischemia [6]. While these data on VTE are more limited, there are still several epidemiological studies that describe psychological stress as a significant risk factor. Perhaps most notably, the Nurses’ Health Study II, consisting of 49,296 women with new-onset VTE and spanning 22 years, showed that exposure to traumatic events was significantly associated with VTE risk [7]. Moreover, women with the most severe posttraumatic stress disorder symptoms had an almost 2-fold greater VTE risk than their unexposed counterparts [7]. Similarly, the Woman Abuse Screening Tool-VTE study, consisting of 997 VTE case-control pairs, found that intimate partner violence was more prevalent among cases than controls (10.9% vs 3.3%) [7]. Prospective observational studies have underscored this association as well; in the longitudinal investigation by von Känel et al. [7], of 271 consecutive patients previously diagnosed with VTE, those who scored higher on the Hospital Anxiety and Depression Scale had a 4-fold greater risk of recurrent VTE than those with milder depressive symptoms. Given the myriad of minority stressors faced by trans persons [8], including violence and discrimination [9], disparities in VTE experienced by trans populations warrant further research beyond strictly hormone therapy.

## 2 Stress-Induced Hypercoagulability

In the 20th century, Harvard physiologist Walter B. Cannon postulated that clotting may have evolved, in part, as an adaptive response to tissue injury and anticipated bleeding during the fight-or-flight response. He demonstrated, in a series of experiments, that cats without adrenal glands exhibited prolonged coagulation times when caged near dogs: “Rapid coagulation may reasonably be considered as an instance of adaptive reaction serviceable to the organism in the injury which may follow the struggle that fear or rage may occasion” [10]. Plainly, hypercoagulability may have evolved, in part, to occur in anticipation of an injury when facing acute stress events.

## 3 Catecholamines and Hypercoagulability

This adaptive stress-induced hypercoagulability acts mainly through 2 hormonal pathways: catecholamine- and glucocorticoid-mediated pathways (Figure). Catecholamines (ie, adrenaline and noradrenaline) promote a prothrombotic state via both primary and secondary hemostasis. In primary hemostasis, stimulation of α_2_- and β_2_-adrenergic receptors on the platelet membrane has been shown *in vitro* to 1) induce platelet aggregation via release of adenosine diphosphate and downstream binding of glycoprotein IIb/IIIa to fibrinogen, and 2) to potentiate the effects of other platelet-aggregating agents such as collagen and thrombin [11]. von Känel et al. [12] systematically reviewed 21 human studies that assessed platelet activity following the infusion of α- and β-sympathomimetic agents. Infusion of epinephrine and norepinephrine led to at least 1 stimulated marker of platelet activation (ie, size, release factors, and surface glycoproteins) in 11 of 15 (73%) and 3 of 6 (50%) studies, respectively. Platelet aggregation was assessed separately by filtragometry, with multiple studies showing dose-dependent responses to epinephrine and norepinephrine [12]. In one study, both adrenaline infusion and a color-word conflict test simulating acute mental stress resulted in statistically significant increases in platelet aggregation, as measured by filtragometry (*P* < .05) [13]. Reversal of epinephrine-induced hyperaggregability with nonselective α-blockade was suggestive of the α_2_-adrenergic receptor’s key role in platelet activation [12]. Endothelial damage caused by inflammatory cytokines during acute stress is another driver of stress-induced platelet activation. Several studies have observed increased plasma levels of inflammatory cytokines (ie, interleukin [IL]-6, tumor necrosis factor-α, IL-2, and IL-10) following acute stress exposure [14]. Furthermore, preexisting stress conditions, including depression, loneliness, and low socioeconomic status (SES), seem to correlate with higher circulating levels of cytokines released during acute stress [14]. In one study of British civil servants, IL-6 levels measured during stressful behavioral tasks differed by employment grade, with adjusted means of 1.30, 1.22, and 1.43 pg/mL in the high- (administrative), intermediate- (professional and senior executive officers), and low- (executive officers, clerical, and office support) grade groups, respectively (*P* = .016) [15]. In the British civil service, employment grade, or the hierarchical classification of a job based on its relative value and required skill set, correlates strongly with income and educational attainment [15].

**Figure:**
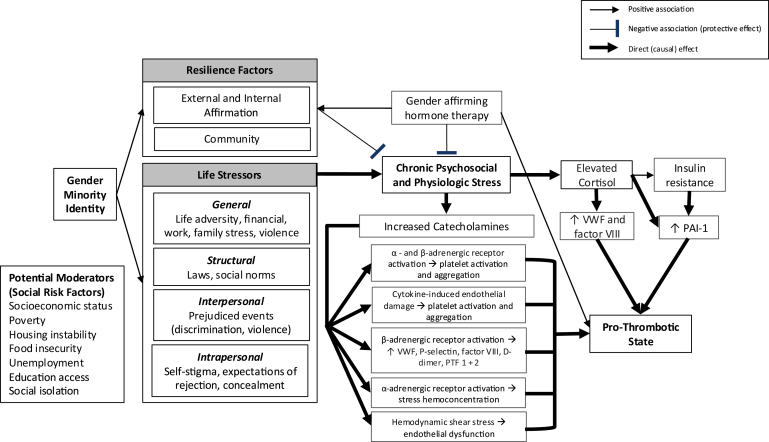
The minority stress theory mapped to thrombotic pathways: gender minority status is associated with both resilience factors and life stressors (thin arrows). Resilience factors include external and internal affirmation and community. Life stressors can be categorized as *general* (life adversity, financial, work, family stress, and violence), *structural* (laws and social norms), *interpersonal* (prejudiced events [discrimination and violence]), and *intrapersonal* (self-stigma, expectations of rejection, and concealment). Resilience factors have a protective effect against chronic psychosocial and physiological stress (blunt arrow). However, life stressors have a direct stimulatory effect on chronic psychosocial and physiological stress (wide arrow). Chronic psychosocial and physiological stress has a direct stimulatory effect on catecholamines (wide arrow), which, in turn, leads to widespread α- and β-adrenergic receptor activation (wide arrows). This results in increased platelet activation and aggregation; increased von Willebrand factor (VWF), P-selectin, factor VIII, D-dimer, and prothrombin fragments (PTF) 1 + 2; stress hemoconcentration; and endothelial dysfunction. These processes directly stimulate a prothrombotic state (wide arrow). Chronic psychosocial and physiological stress simultaneously has a direct stimulatory effect on cortisol (wide arrow), which, in turn, leads to increased VWF and factor VIII (wide arrows), and increased plasminogen activator inhibitor-1 (PAI-1). These 2 processes also directly stimulate a prothrombotic state (wide arrows). Elevated cortisol is associated with insulin resistance (thin arrow), which also leads to increased PAI-1 (wide arrow). While gender affirming hormone therapy is associated with a prothrombotic state (thin arrow), it is also associated with resilience factors (thin arrow) and has a protective effect (blunt arrow) against chronic psychosocial and physiological stress. Social risk factors (socioeconomic status, poverty, housing instability, food insecurity, unemployment, education access, and social isolation) are potential moderators of these thrombotic pathways.

In secondary hemostasis, catecholamines promote a prothrombotic state via 1) activation of β-adrenergic receptors on endothelial vasculature, 2) stress hemoconcentration, and 3) endothelial dysfunction [16]. β-adrenergic receptor activation initiates an intracellular signaling cascade that causes a surge in cyclic adenosine monophosphate, leading to sustained release of Weibel–Palade bodies and their procoagulant contents (von Willebrand factor [VWF], P-selectin, and factor [F]VIII), and, in turn, elevating downstream markers of hypercoagulability, such as D-dimer and prothrombin fragments 1 + 2 [16]. Gatica et al. [16] systematically reviewed the effects of adrenergic receptor-targeting drugs on coagulation parameters. Overall, they found that a prothrombotic state was induced by β-adrenergic receptor agonist stimulation, and a profibrinolytic state was induced by α_1_- and β-adrenergic receptor antagonist stimulation. For example, D-dimer increased with β-adrenergic receptor agonists such as salbutamol (from 0.26 [mean] ± 0.04 [SE] mg/L to 0.73 [mean] ± 0.25 [SE] mg/L; *P* < .001) and decreased with α_1_- and β-adrenergic receptor blockers such as carvedilol (from 428 [mean] ± 306 [SD] μg/dL to 218 [mean] ± 164 [SD] μg/dL; *P* < .01) [17,18].

Interestingly, extended adrenergic receptor stimulation had effects similar to those of short-term stimulation. This suggests that chronic catecholamine stimulation, as in the setting of chronic stress [19], may yield procoagulant effects comparable to those of acute stimulation. Indeed, observational studies looking at elderly Alzheimer caregivers, exhausted teachers, and overworked accountants are highly suggestive of an association between chronic mental stress and hypercoagulability [16]. In a study by von Känel et al. [20] of spousal Alzheimer’s disease caregivers, D-dimer was significantly higher in the caregiver group (688 ± 575 ng/mL vs 406 ± 157 ng/mL; *P* = .02), even after adjusting for body mass index, smoking status, and hypercholesterolemia. A subsequent study showed that the number of negative life events experienced by spousal Alzheimer’s disease caregivers accounted for an additional 13% of variance in allostatic load, as measured by increased procoagulability scores from baseline, even after controlling for age and smoking status (*P* = .01) [21].

Stress hemoconcentration occurs when α-adrenergic vasoconstriction leads to plasma leakage into the interstitium, thereby increasing the viscosity of intravascular fluid and the concentration of procoagulant proteins within the vasculature [22]. Catecholamine release also elicits hemodynamic effects (ie, hypertension, tachycardia, and increased cardiac output) that contribute to shear forces, leading to endothelial dysfunction and downstream activation of the coagulation cascade [23]. Effectively constituting Virchow’s classic triad of hypercoagulability, blood stasis, and vascular endothelial injury, these 3 mechanisms collectively increase the risk of thrombosis during both acute and chronic sympathetic stimulation.

## 4 Cortisol, Insulin Resistance, and Hypercoagulability

In the glucocorticoid-mediated pathway, cortisol, under the control of the hypothalamic-pituitary-adrenal axis, is released by the adrenal glands in response to stress. Whereas catecholamines appear to exert procoagulant effects in both acute and chronic stress states, cortisol’s effects on the coagulation system seem to have a bimodal distribution [23]. Studies have shown that the acute administration of steroids may have a protective effect against pulmonary embolism in high-risk trauma patients. Yet, long-term glucocorticoid treatment is known to be associated with an increased incidence of VTE [23]. A large population study by Johannesdottir et al. [24] of 38,765 VTE cases demonstrated increased adjusted incidence rate ratios among current (2.31; 95% CI: 2.18, 2.45), new (3.06; 95% CI: 2.77, 3.38), continuing (2.02; 95% CI: 1.88, 2.17), and recent (1.18; 95% CI: 1.10, 1.26) systemic glucocorticoid users. Furthermore, the increased incidence rate ratios appeared to be dose-dependent, correlating with prednisolone-equivalent cumulative doses ranging from <10 to >1000 to 2000 mg [24]. Similar to catecholamines, glucocorticoid-induced hypercoagulability is mediated by elevated plasma concentrations of VWF and FVIII [23]. Cortisol is also a potent enhancer of the plasminogen activator inhibitor-1 (PAI-1) promoter, thereby upregulating PAI-1 transcription [22]. In one study, glucocorticoid treatment significantly increased PAI-1 secretion in adipose tissue (from 1.9 [mean] ± 0.2 [SE] ng/g triglycerides to 3.5 [mean] ± 0.5 [SE] ng/g triglycerides; *P* = .01) [25]. Given that PAI-1 is an extremely potent inhibitor of tissue-type plasminogen activator, increased PAI-1 transcription leads to a hypercoagulable state [26].

Insulin also plays an important role in PAI-1 transcription. Anxiolytic yet maladaptive behaviors, often seen in chronic stress, such as carbohydrate craving and alcohol abuse, contribute to hyperinsulinemia. Indeed, a high-carbohydrate diet and a sedentary lifestyle lead to insulin hypersecretion [22]. Like cortisol, insulin is a potent enhancer of the PAI-1 promoter [27]. It also indirectly increases PAI-1 transcription via its lipogenic properties and the excess formation of adipocytes. These fat cells can produce tumor necrosis factor-α, an inflammatory cytokine that inhibits insulin receptor signaling [22]. As a result, insulin resistance develops over time, and further insulin secretion occurs as a compensatory response. The end result is a positive feedback loop in which postprandial hyperinsulinemia leads to obesity, which in turn leads to insulin resistance, which in turn leads to further hyperinsulinemia, effectively driving continuous PAI-1 transcription [22]. Another mechanism of obesity-related hypercoagulability is via aromatase activity in adipose tissue, which leads to the synthesis of estradiol from testosterone and estrone from androstenedione substrates [28]. Estrogens and their metabolites are known to exert prothrombotic effects [29].

Hypercortisolemia can also cause insulin resistance, thereby amplifying this positive feedback loop. Through various mechanisms, including impaired insulin receptor signaling, pancreatic β-cell dysfunction, inflammatory cytokine production, adipose tissue accumulation, and chronic hyperglycemia, elevated cortisol levels can lead to a constellation of metabolic derangements known as metabolic syndrome (MetS) [30,31]. Insulin resistance is a hallmark of MetS and can drive its progression to overt diabetes. Thus, cortisol and insulin may act synergistically to promote both PAI-1 transcription and adipose tissue aromatase activity. Trans adults are at higher risk of developing MetS compared with their cisgender peers. In fact, a 2016 study of 5135 transgender veterans demonstrated a higher likelihood of obesity compared with cisgender controls (aOR, 1.58 [95% CI: 1.48, 1.70]) [32]. This association is likely multifactorial, with disparities in healthy lifestyle behaviors, increased rates of disordered eating, increased rates of depression and anxiety, body compositional changes associated with gender affirming hormone therapy (GAHT), and socioeconomic and healthcare barriers to accessing affirming care all reasonably contributing to the higher prevalence of obesity in trans people [32]. While data on the association between obesity and thrombosis specifically in trans persons are limited, obesity is a well-established risk factor of VTE in the general population. In fact, the Tromsø Study estimated that almost one-fourth of all VTE events in Norway can be attributed to being overweight and obese [33]. Further research is needed to better quantify the comparative impacts of catecholamines and glucocorticoids on stress-induced hypercoagulability.

## 5 Thrombosis and Stress in Trans Persons

These stress-induced prothrombotic pathways have not been described in trans populations. While exogenous hormone therapy must be considered in understanding the thrombotic risk of trans persons, there may be additional factors in explaining VTE disparities in trans populations. According to a 2020 systematic review of 11 studies reporting VTE rates among transgender patients, 6 in cisgender female patients, and 5 in cisgender male patients, the incidence of VTE was higher in transgender patients assigned male at birth compared with those assigned female at birth (42.8 vs 10.8 per 10,000 patient years; *P* = .02) [34]. Although the review reported VTE incidences in patients assigned male at birth on feminizing GAHT that were similar or slightly higher than in cisgender females on estrogen hormone replacement therapy, some reports have suggested up to a 20-fold increase in VTE incidence compared with nonmedicated cisgender females [34]. Patients assigned female at birth on masculinizing GAHT, however, appear to experience VTE rates similar to cisgender males on testosterone hormone replacement therapy [34]. While it appears that these data suggest exogenous hormone therapy as a primary driver of VTE in transgender persons, it is worth noting that the study was underpowered to assess the impact of different estrogen formulations and delivery methods. Of note, the predominant estrogen formulation in oral contraceptive pills (ethinyl estradiol) is significantly more thrombogenic than that in feminizing GAHT (17-β estradiol) [35].

We remain committed to our argument that additional trans-specific factors warrant further evaluation, given the complex interactions of VTE pathogenesis. Minority stress theory may be an additional factor in explaining VTE disparities in trans populations. The minority stress theory posits that both proximal (internalized transphobia, negative expectations, hypervigilance, and concealment of gender identity) and distal (gender nonaffirmation and discrimination, rejection, and victimization based on gender identity) stressors subject trans people to unique and higher stress levels, which, in turn, predispose them to health issues through psychological and biological mechanisms and the shaping of maladaptive behaviors [36]. While there is increasing interest in the effects of stress on various physical, mental, and behavioral health outcomes, there is limited research exploring the association between stress and VTE in trans populations.

## 6 Social and Socioeconomic Risk Factors as Proxies For Chronic Stress

Opportunities to measure stress at a population level are overall limited. Social and socioeconomic risk factors can theoretically be assessed as proxies for chronic stress [37]. While social determinants of health are defined by the World Health Organization as “the conditions in which people are born, grow, live, work, and age,” social risk factors are adverse social conditions, such as low SES, social isolation, and housing instability, that arise as a consequence of inequitable social determinants of health exposures and are associated with poor health outcomes [38]. The likelihood of experiencing stress in individuals with social risk factors is disproportionately higher. While the relationship between SES and thrombosis is not clearly defined, some epidemiological studies have examined the associations between different measures of SES (ie, education, occupation, and social class) and blood coagulation factors [39]. Most notably, a large meta-analysis demonstrated that unemployment and lower education were associated with higher fibrinogen levels, even after controlling for various CVD risk factors [40]. It is worth noting that the studies included in this meta-analysis [40] presumably evaluated cisgender men and women. To our knowledge, there are no studies assessing SES and thrombosis risk in trans persons specifically.

There is also mounting research looking at intersectional minority stress, noting that SES may play a moderating role [41]. Because trans people face significant socioeconomic disparities, it is important to contextualize low SES, arguably the most prevalent social risk factor, within the minority stress theory for this population. The relationship between social risk factors and health, in particular, is synergistic and bidirectional. While a high burden of social risk factors can contribute to worse health outcomes, issues related to one’s health can simultaneously exacerbate existing social risk factors. Therefore, trans people often find themselves trapped in a vicious cycle of both health and socioeconomic decline. Research on social risk factors and SES in this understudied population remains largely absent.

Trans populations face significant socioeconomic disparities due to factors such as workplace discrimination, marginalization across the lifespan, and legal barriers to socioeconomic equality [42]. According to data from the TransPop study, a greater proportion of transgender people had an education level of high school or less (48.6% [95% CI: 35.7%, 61.7%] vs 33.9% [95% CI: 29.3%, 38.8%]), received food stamps or Women, Infants, and Children (WIC) benefits (a special supplemental nutrition program in the U.S. for low-income women, infants, and children; 36.0% [95% CI: 22.4%, 52.3%] vs 10.4% [95% CI: 7.6%, 14.0%]), and met criteria for poverty (39.3% [95% CI: 26.6%, 53.5%] vs 12.2% [95% CI: 9.1%, 16.3%]) than cisgender people [4]. Poverty rates among trans people of color are particularly alarming. For example, almost half of Latin trans adults (48%) and approximately 4 out of 10 Black trans adults (39%) are living in poverty [43]. According to the 2022 U.S. Transgender Survey, more than one-third (34%) of 92,329 respondents were experiencing poverty, and nearly one-third (30%) had experienced homelessness in their lifetime [9]. Furthermore, food insecurity, housing instability, and unemployment rates in the transgender population have all been worsened by the COVID-19 pandemic [43]. It is no surprise then that trans people fare more poorly with a wide range of health, functioning, and quality-of-life aspects.

## 7 Future Research Directions

Researchers and clinicians should assess the role of low SES and other social risk factors in perpetuating stress and, by extension, exacerbating thrombosis risk in trans persons. This is especially relevant for those on feminizing hormone therapy, as estrogen can have a compounding hypercoagulable effect in the setting of already high levels of circulating catecholamines and cortisol. Recently, there have been studies on CVD suggesting that testosterone may also be a procoagulant [44,45]. As GAHT is an evidence-based treatment for gender dysphoria, screening for social risk factors should occur both prior to initiation of hormones and during routine monitoring while on hormones. While GAHT may increase the risk of VTE by altering procoagulant protein levels, it may also have an indirect protective effect against thrombosis by alleviating chronic stress (Figure).

Future research efforts should therefore evaluate SES in relation to thrombosis in trans persons with and without hormone therapy. Findings may have screening implications for social risk factors in trans patients on GAHT, as well as encourage medical providers to better address the health-related social needs (HRSN) of this vulnerable population. Furthermore, HRSN screening at both intake and follow-up could potentially allow for improved thrombosis risk stratification of trans patients. Namely, patients with a higher burden of HRSN and perceived stress may benefit from discussions about more frequent monitoring, alternative hormone delivery methods or formulations (ie, transdermal estrogens are less thrombotic than oral estrogens [46]; conjugated equine estrogens are significantly more thrombogenic than 17-β estradiol [47]), or even the utility of prophylactic anticoagulation. Finally, identification of the presence of social risk factors and/or the absence of resilience factors in this population (who already experience minority stress at baseline) can aid health care providers in implementing interventions that reflect both social needs-informed and social needs-targeted care.
